# Bayesian Inference of the Evolution of HBV/E

**DOI:** 10.1371/journal.pone.0081690

**Published:** 2013-11-29

**Authors:** Iris E. Andernach, Oliver E. Hunewald, Claude P. Muller

**Affiliations:** Institute of Immunology, Centre de Recherche Public de la Santé/Laboratoire National de Santé, Luxembourg, Luxembourg; Columbia University, United States of America

## Abstract

Despite its wide spread and high prevalence in sub-Saharan Africa, hepatitis B virus genotype E (HBV/E) has a surprisingly low genetic diversity, indicating an only recent emergence of this genotype in the general African population. Here, we performed extensive phylogeographic analyses, including Bayesian MCMC modeling. Our results indicate a mutation rate of 1.9×10^−4^ substitutions per site and year (s/s/y) and confirm a recent emergence of HBV/E, most likely within the last 130 years, and only after the transatlantic slave-trade had come to an end. Our analyses suggest that HBV/E originated from the area of Nigeria, before rapidly spreading throughout sub-Saharan Africa. Interestingly, viral strains found in Haiti seem to be the result of multiple introductions only in the second half of the 20th century, corroborating an absence of a significant number of HBV/E strains in West Africa several centuries ago. Our results confirm that the hyperendemicity of HBV(E) in today's Africa is a recent phenomenon and likely the result of dramatic changes in the routes of viral transmission in a relatively recent past.

## Introduction

Hepatitis B virus (HBV), a major public health burden, is a partially double-stranded circular DNA virus of ∼3.2 kb. Due to its small size, HBV DNA has a compact organization with four overlapping open reading frames (ORFs), coding for the hepatitis B surface antigen HBsAg (coded by the preS1/preS2/S ORF), the polymerase (P ORF), the X protein (X ORF) and the hepatitis B e and core antigen (preC/C ORF) [1].

As the viral polymerase lacks proofreading capability, HBV has evolved into at least 8 recognized genotypes A-H [2], [3] and a tentative genotype I [4], [5]. Recently a tenth genotype J has been proposed in strains from a Japanese patient [6]. With the exception of HBV/E, G and H, genotypes are divided into subgenotypes, with more or less distinct geographic distributions [2], [3], [4], [5], [7], [8], [9], [10], [11]. In Africa one of the three genotypes E, A and D predominates depending on the region. While genotype D is the most prevalent variant in Northern Africa, genotype A prevails in East and South Africa. Except for Cameroon, where genotype A is dominant, genotype E is highly endemic in most of sub-Saharan Africa. Despite its high prevalence and wide geographic spread throughout large parts of Africa, HBV/E has a surprisingly low mean genetic diversity of only 1.75% over the full-length genome, in contrast to 4% diversity for the African HBV/A strains [11]. It has been suggested that it would take only about 200 years for the genetic diversity of HBV/E to develop [12], [13], while recent studies indicate an even more recent emergence of HBV/E [14], [15]. African slaves that were force-migrated to the Americas during the transatlantic slave trade between the 16^th^ and 19^th^ century were expected to have disseminated HBV/E in the New World, but the absence of significant numbers of genotype E strains outside of Africa supports a more recent emergence of this genotype in Africa [12]. To understand the evolution of the virus, a number of studies have been performed to assess the nucleotide substitution rate of HBV. However, because of differences in the computational approach as well as the dataset, large differences of nucleotide substitution rates were observed, ranging from ∼10^−4^ to 10^−5^ substitutions per site and year (s/s/y) [16], [17], [18], [19], [20], [21]. As most of these studies were based on repeated sampling in the same chronic carriers of the virus or on viral strains collected from mother child pairs, the observed substitution rates are largely indicative of short evolutionary rates and may not necessarily reflect general longer term evolutionary rates. A recent study estimating HBV mutation rates on a selected dataset used Bayesian Markov-Chain-Monte-Carlo (MCMC) analyses and revealed a relatively high substitution rate of 3.7×10^−4^ to 7.70^−4^ s/s/y on the full-length genome [22]. A substitution rate of 3.2×10^−4^ was furthermore reported for a small HBV/E dataset [15]. All of these studies indicate an only recent introduction and a short time of evolution of HBV/E in the general population of sub-Saharan Africa.

Recently, a study investigated the long-term evolution of HBV worldwide on a selected HBV S-gene dataset, including all genotypes and subgenotypes [23]. The authors linked the global spread of HBV to human migratory patterns to estimate a global evolution during the past 34,000 years and found a long-term substitution rate of 2.2×10^−6^ s/s/y on the S-gene which is almost 100 times slower than previous estimates. Based on this mutation rate, the tMRCA of genotype E was estimated to be 6000 years.

In our study, we address these apparent discrepancies and provide evidence that HBV/E has indeed only recently been introduced into the general West African population. We performed phylogeographic analyses, including Bayesian MCMC modeling on extensive HBV/E full-length genome and S-gene datasets, in order to infer the timescale and dynamics of evolution of HBV/E. Our results confirm an only recent emergence of HBV/E, most likely within the last 130 years, and rapid expansion of HBV/E in sub-Saharan Africa most probably due to dramatic changes in routes of viral transmission.

## Materials and Methods

### Sequences

Non-recombinant sequences of HBV genotype E for which the country and year of sampling was available were obtained from NCBI (accessed December 2011). Recombinant strains were excluded using jpHMM [24] and the RDP, GENECONV and MaxChi options of RDPv.3.44 [25], [26], [27]. The dataset included 167 full-length genome sequences and a total of 454 S-gene sequences with the accession numbers AB194947, AB194948, AB205188-AB205192, AM494689-AM494715, AM494717, AY738147, FN545821-FN545824, FN545827, FN545841, FN545842, GQ161755-GQ161766, GQ161768-GQ161774, GQ161776-GQ161787, GQ161789-GQ161805, GQ161807-GQ161845, GQ161847-GQ161812, GQ161814-GQ161836, HM363565-HM363611 (full-length) and AB194954, AB194955, AB205323-AB205329, AF323617-AF323619, AF323620, AF323621-AF323636, AJ604932-AJ605031, AM494719-AM494723, AM494725, AM494727, AM494730-AM494734, AM494737-AM494741, AM494743-AM494748, AM494751-AM494753, AM494832, FJ692540, FJ692544-FJ692548, FJ692550-FJ692553, FN547192-FN547199, FN547202-FN547205, FN547207, FN547209-FN547215, FN547217, FN547218, FN547220, FN547221, FN547225-FN547227, FN547229, FN547230, FN547232, FN547234, FN547236, FN547237, FN547239, FN547240, FN547248, FN547251-FN547253, FN547255-FN547257, FN547259, FN547261, FN547263-FN547272, FN547274-FN547277, FN547279, FN547282-FN547288, FN547292-FN547294, FN547297-FN547299, FN547304, FN547310, FN547333, FN547339, FN547347, FN547356, FN547358, FN547359, FN547362, GQ161775, GQ161806, GQ161839, GQ161842, GQ161844, GQ161846, HM195104-HM195106, HM195108-HM195113, HQ385227, HQ385235, HQ385236, HQ385238, HQ385239, HQ385241, HQ385242, HQ385245, HQ385246, HQ385248-HQ385257, HQ385260-HQ385265, HQ385267 (S-gene only). Origins and sampling dates of the analyzed HBV sequences are summarized in Table 1.

**Table 1:** Origin and sampling date of analyzed HBV genotype S full-length and S-gene sequences.

		No. analyzed sequences	
Country	Year	Full length	S-gene
Angola	2007	/	9
Benin	2001	/	13
Burkina Faso	2001	/	10
Cameroon	2007	2	11
	2006	1	1
	2005	/	3
	2002	/	19
	1994	2	4
Central African Republic	2004	28	55
Congo DRC	2001	/	3
	1998	1	1
Ghana	2000	5	15
Guinea	2006	77	79
Haiti	2006	/	10
Mali	2002	/	18
Nigeria	2007	47	47
	2006	4	40
	2005	/	33
	2001	/	15
	1998	/	8
	1997	/	9
	1996	/	3
Sudan	2009	/	26
Togo	2001	/	22
Total		167	454

The full-length and S-gene sequence sets were aligned using MAFFT v6 for Windows with the L-INS-i option [28], [29].

### Phylogenetic and phylogeographic analyses

Model estimation was performed for both the HBV/E full-length and S-gene datasets, using Topali v2 [30]. Based on the Akaike information criteria obtained the general time-reversible model with four gamma categories and invariant sites (GTR+G+I) was selected for each dataset. In addition, the less complex symmetrical (SYM) and transversion (TVM) models were tested on the S-gene alignment. The analyses were carried out using the BEAST v.1.6.2 software package [31]. Parameter settings were defined using BEAUTi v.1.6.2 [31] with subsequent manual adjustments and analyses were performed with BEAST. As BEAST uses the Bayesian Markov-Chain-Monte-Carlo approach to sample states from a probability distribution and samples the root position of the phylogeny along with the rest of the nodes, no out-group strains are necessary for analysis. Furthermore, the inclusion of the sampling year of the individual strains allows a time measured inference with BEAST. The HBV/E sequence sets were analyzed with a strict or relaxed molecular clock, using constant size, exponential and expansion growth tree priors. Additionally, geographical information was included as a “state” location parameter for a subset of parameterized runs. For each run all states before convergence and at least 10% of states, were discarded as burn-in. Each parameterized run was repeated to reach effective sample sizes (ESS) greater than 200. Runs were assessed using Tracer [31] and merged using LogCombiner v.1.6.2 [31]. The most applicable parameterized merged runs were selected based on the highest Bayes Factors, as calculated by Tracer.

Tree calculation of the resulting output files was performed with TreeAnnotator v.1.6.2 [31] and visualization with FigTree v1.3.1 (http://tree.bio.ed.ac.uk/software/figtree). Geographic spread analysis of HBV/E was performed on S-gene tree files including geographical information. The SPREAD v1.0.3 [32] output file was processed using Google Earth v6.2.1 (Google Inc.) and visualized using Map Resources (www.mapresources.com) and Microsoft Power Point (Microsoft Corporation). Longitude and latitude data of the individual country midpoints were included in the analyses as geographical coordinates. Bayesian skyline analyses were performed in BEAST, using the Bayesian skyline coalescent of BEAUTi.

### Median-joining network (MJN)

A median joining network of the 454 S-gene strains was constructed using Network v4.610 and visualized using Network Publisher [33] (Fluxus Technology, Germany). Briefly, MJN were constructed starting from minimum spanning trees that were combined within a single network. Aiming at parsimony, consensus sequences of 3 mutually close sequences were subsequently added at a time. These so called median vectors can biologically be interpreted as extinct ancestral sequences. This median operation then resulted in the most optimal network, reflecting the shortest possible distances between the individual nodes [33].

## Results

### Substitution rates and tMRCA

To estimate the time of evolution from a most recent common ancestor (tMRCA) and assess the nucleotide substitution rates, we performed Bayesian coalescent analyses on 167 HBV/E full-length and 454 S-gene strains. For both sequence sets the Bayes Factors (BF) significantly favored a Relaxed Molecular Clock model, with the Uncorrelated Exponential Clock having a higher BF than the Uncorrelated Lognormal Clock. Furthermore the Expansion Growth Coalescent with a UPGMA generated starting tree was favored.

Based on these parameters the substitution rate of the full-length HBV/E was calculated to be 1.86×10^−4^ substitutions per site and year (s/s/y) with a confidence interval (95% HDP) ranging from 1.91×10^−5^ to 3.79×10^−4^ and a median tMRCA of 130 years (mean tMRCA of 174 years; 95% HPD: 36–441 years). When including the geographic “state” parameter as a discrete phylogeographic inference, the substitution rate changed to 2.23×10^−4^ s/s/y and a median tMRCA of 116 years (mean 145 years; 95% HPD: 37–336 years), indicating an influence of the geographic “state” parameter in the analysis. While inclusion of this parameter is a prerequisite for visualization of the phylogeographic data, the resulting time estimations would have to be considered with caution. Since today's geographic boundaries were not present at the supposed time of the spread and discrete geographic “states” have changed over time, one would expect the tMRCA without the geographic parameter to be more reliable with respect to time of evolution of HBV/E. Furthermore, calculations were performed using median values, as this would reduce the influence of rare outliers in the analyses.

Analyzing the S-gene sequences, the mutation rate of 1.9×10^−4^ s/s/y, as calculated for the full-length genome, would correspond to a median tMRCA of 71 years (mean 73 years; 95% HPD: 54–97) and 52 years (mean 53 years; 95% HPD: 39–71 years) when including the geographic “state” parameter. Based on a tMRCA of 130 years, as calculated for the full-length genome the mutation rate for the S-gene strains would correspond to about 7×10^−5^ s/s/y, (126 years tMRCA), while inclusion of the geographic parameter would increase the timeframe to about 156 years tMRCA.

### Geographic distribution of HBV/E

**Figure 1:**
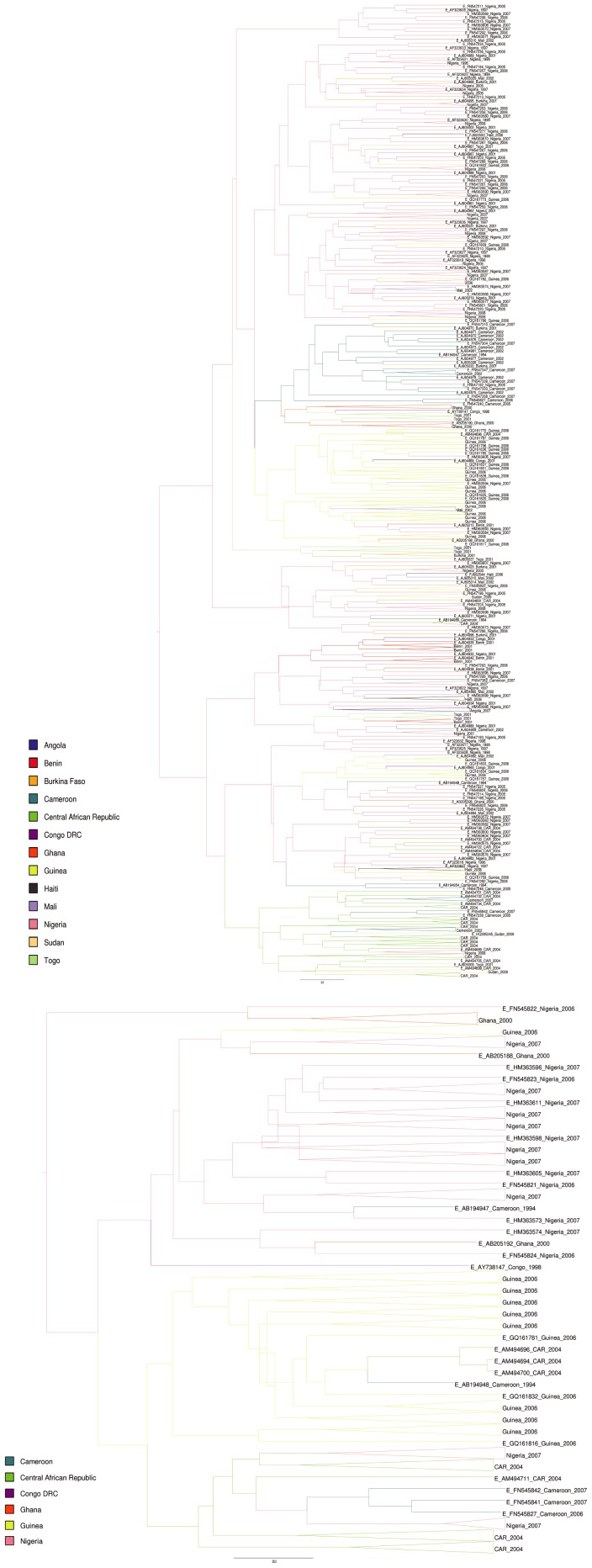
Phylogenetic analyses of all available HBV/E S-gene and full-length sequences. Analyses of S-gene (a) and full-length sequences (b) were performed using the GTR+G+I model with geographic information. Branching and roots of strains from individual countries are indicated by colors. Clusters with strains sampled in the same country and during the same year are collapsed.

**Figure 2:**
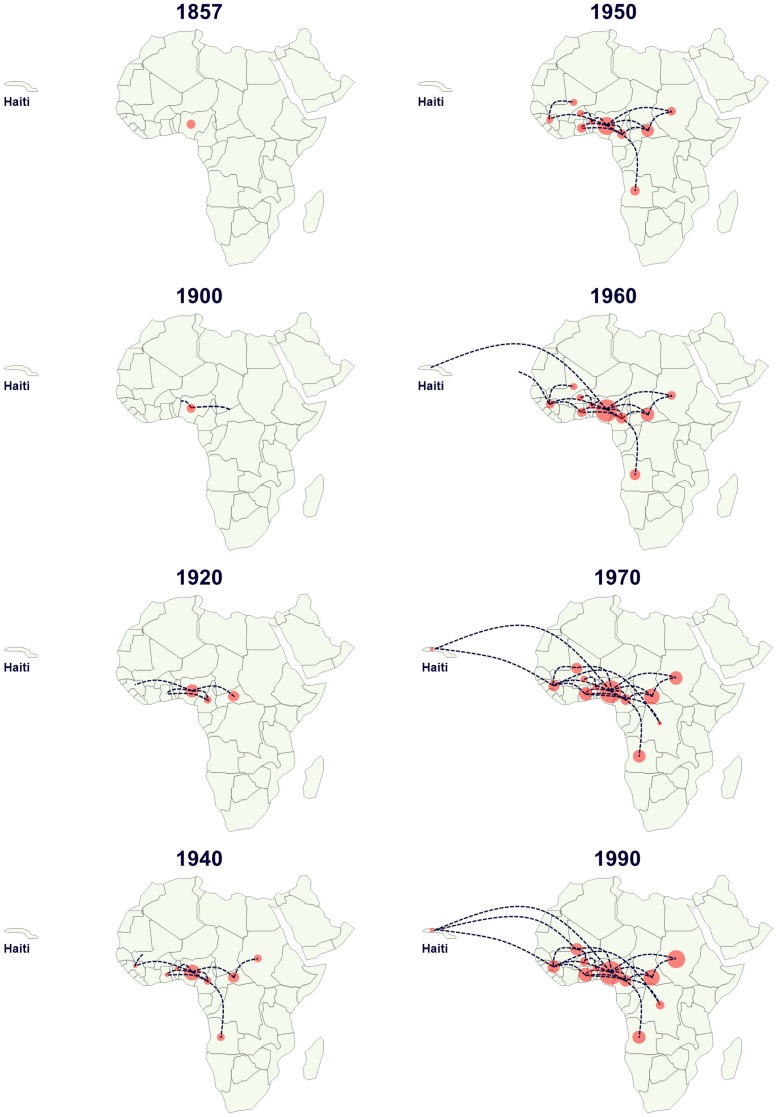
Phylogeographic spread of HBV/E. The snapshots represent the geographic and temporal spread of HBV/E for which at least the S-gene and the spatial and temporal sampling information were available. A mutation rate of 7×10^−5^ s/s/y with the GTR+G+I model with geographic information was used. Spread analysis by the SPREAD software was visualized using Map Resources.

Phylogeographic analyses of both the S-gene and full-length dataset showed that HBV/E strains formed several clusters. Interestingly, strains from individual countries did not necessarily cluster together, while strains sampled within one country at different time points could be found in the same, but also in different clusters (Figures 1a and b). The analysis furthermore revealed a putative origin of HBV/E in the area of Nigeria. This is supported by spatial phylogenetic reconstruction that revealed a clustering of HBV/E primarily in the region of Nigeria and a putative spread along the West African coast and to Angola, Congo DRC and the Sudan (Figure 2).

### HBV/E population growth

**Figure 3:**
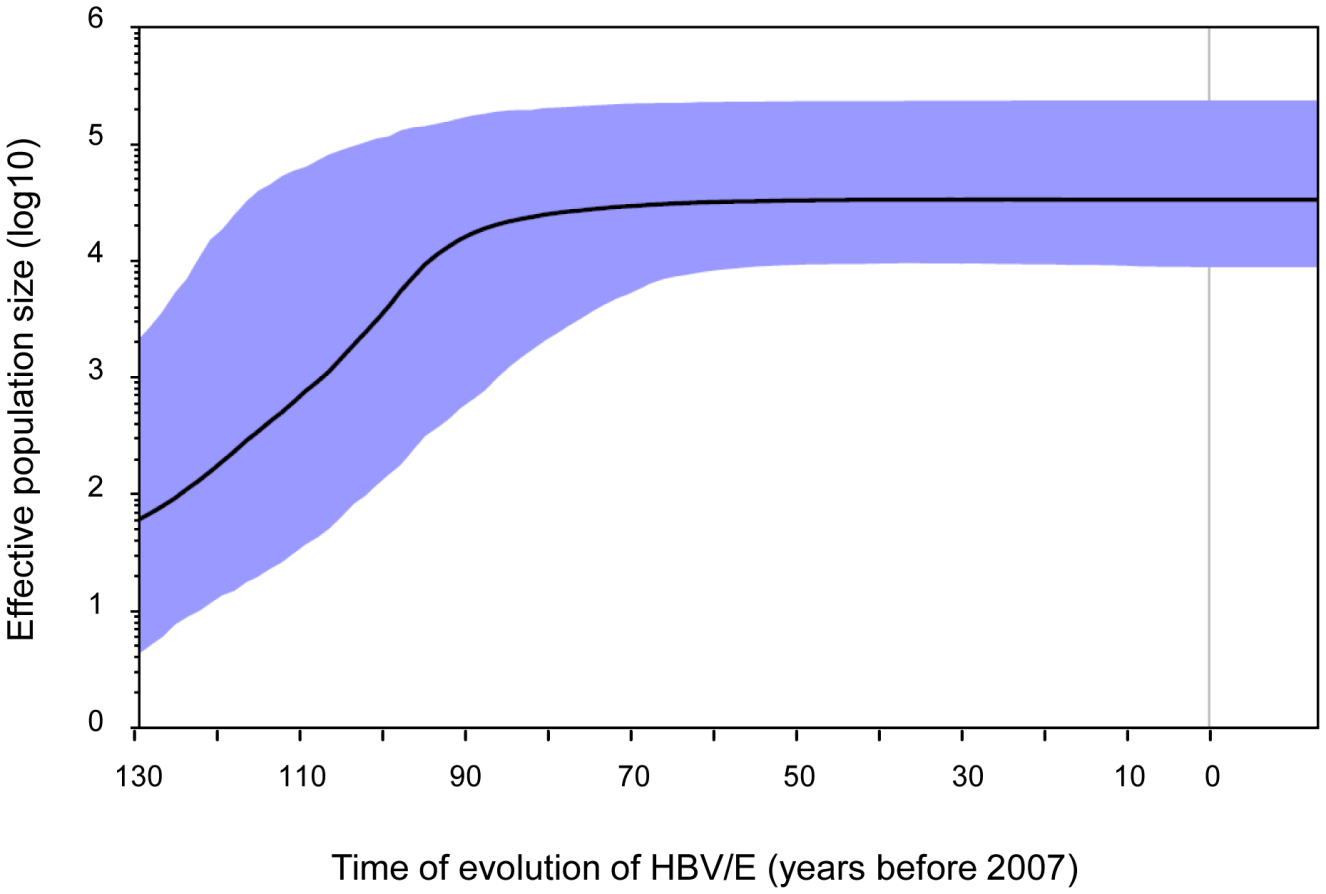
Bayesian skyline plot showing the epidemic history of the HBV/E S-gene dataset. The plot indicates the median estimate of the effective population size, with the 95% highest posterior density indicated in blue. The applied timeframe ranges between the most recent sampling date and the calculated 130 years of evolution from the most recent common ancestor (MRCA), as calculated in the HBV/E full-length analysis.

**Figure 4:**
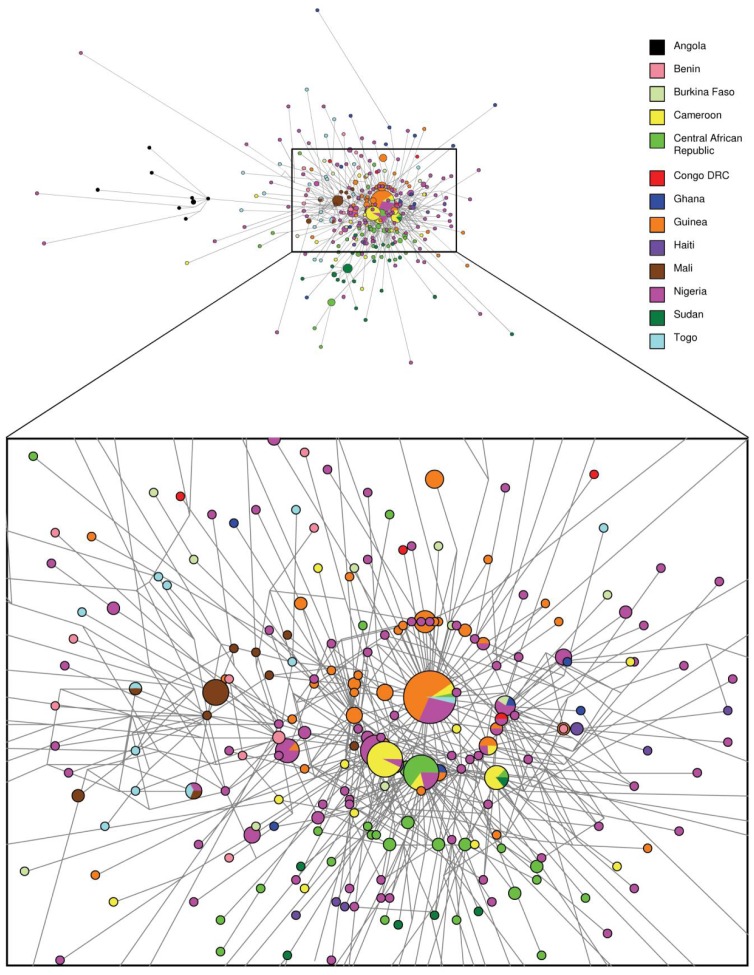
Median Joining Network of HBV/E S-gene sequences. Pie charts represent sequence variants at the nodes, with colors indicating the country of sampling of individual sequences, the sizes reflecting the frequencies of the corresponding variants.

The low genetic diversity and wide spread of HBV/E variants in sub-Saharan Africa suggests a rapid and recent increase in the number of HBV/E infections. Indeed, Bayesian skyline analyses of S-gene strains show a rapid population expansion over time. Based on a mutation rate of 7×10^−5^ s/s/y, corresponding to approximately 130 years of evolution from a MRCA, the most significant expansion of HBV/E infections would have occurred from the 1880s until the 1930s (Figure 3). This rapid expansion is furthermore supported by the star-like topology of the median joining network (MJN) analysis (Figure 4). The central clustering of strains originating most prominently from Nigeria and Guinea, countries that are several thousand kilometers apart, indicate a recent and rapid spread of HBV/E. Haiti, in contrast, does not show a central clustering but rather monophyletic clades with a tMRCA of 62.4 and 55.9 years.

## Discussion

We performed extensive phylogenetic as well as phylogeographic analyses to characterize the origin and evolution of HBV/E. Such studies have obvious limitations due to sampling bias, since sampling is necessarily fragmented and incomplete. Nevertheless, under these assumptions, and including all available HBV/E strains, our analyses suggest a putative origin of HBV/E in what is today Nigeria, irrespective of whether the S-gene or the full-length dataset was analyzed. This finding is robust even when the oldest sequences from Nigeria are removed (data not shown). From this region the virus seems to have spread along the West African coast in the region from Guinea to the Central African Republic and subsequently towards Eastern and Southern countries, such as Sudan, Angola and the Democratic Republic of the Congo (Figure 1).

Median-joining network (MJN) analysis of this pool of genetically similar or even identical viral variants, which circulate all over sub-Saharan Africa, reveals a star-like topology, indicating a centralized origin of HBV/E (Figure 4). While the MJN analysis cannot give information on the temporal spread of HBV in Africa, the tight clustering of strains from distant countries several thousand kilometers apart, with sequences from Nigeria and Guinea dominating in the center of the MJN, further support a recent and rapid spread in countries along the West African coast. Indeed, Bayesian analyses revealed a median time of evolution from a most recent common ancestor (tMRCA) of 130 years, with a substitution rate of 1.9×10^−4^ substitutions per site and year (s/s/y).

In contrast to most other studies these values were based on an approach with limited priors to avoid unnecessary biases. In particular, we did not assume a specific time of evolution or mutation rate. This together with the limited sampling period (13 years) would explain to a large extent the high confidence interval (36–441 years) of the 130 years tMRCA.

While the analyses of both viral phylogeny and migration patterns with BEAST have been shown to reduce the analytical bias, as compared to other methods, it was nevertheless also found to reduce the statistical power of the output [34] further increasing the confidence interval.

Some authors have suggested that the tip-date approach using BEAST may not be appropriate for the analysis of HBV. While alternative approaches such as parsimony analyses, increase the statistical power of the observed results, they do not provide information on the probability of the obtained results or provide time estimates of the phylogeny.

Although the analysis of HBV/E is limited by the typical low genetic diversity of this genotype as well as the short sampling period, the BEAST software, allowing for repeated calculations, gave an Effective Sample Size (ESS) value that confirmed that the analysis was statistically robust. This ESS value is a quality measure of the stochastical analysis and expresses the number of effective independent draws from the posterior distribution that the Markov-Chain is equivalent to. The uncertainty due to limitations of the available sequences is difficult to overcome by computational methods without the possibility of a deeper calibration with older viral strains. However, since HBV is transmitted by chronic carriers from generation to generation, the absence of a large number of HBV/E strains outside of Africa provides additional independent clues. As the spread of pathogens is closely linked to their host, African slaves that were force-migrated to the Americas from the 17^th^ to the early 19^th^ century during the transatlantic slave trade would have disseminated African HBV strains present at that time in the New World. Nevertheless, with the exception of single sporadic cases with links to Africa, HBV/E has not been found outside of Africa, corroborating an only recent emergence of this genotype (reviewed in [11]). Indeed studies in Haiti and Martinique, with large populations of descendants of African slaves, revealed surprisingly low prevalences of HBV/E: While our study on HBV from Haiti [12] revealed a HBV/E prevalence of only 6.1%, this genotype was even completely absent in Martinican patients [35], as the rare HBV/E strains observed in that study were attributed to African patients. Thus, the apparent absence of HBV/E in the descendants of African slaves clearly indicates that this genotype cannot have been present in the West African population when and where the slaves were rounded up.

This is furthermore supported by a recent study on HBV subgenotype A1 from Africa [36] that uses phylogeographic analyses based on the Bayesian method implemented in BEAST to trace the spread of this subgenotype in and out of Africa. While not directly calculating the evolutionary time points for this genotype, the authors demonstrate that the spread of A1 correlates well with historical trade- and slave routes between the 9^th^ and 19^th^ century and confirm our earlier inference [12] of multiple early introductions into Haiti of distinct A1 strains that continued to spread in Haiti's population [36]. If genotype E had a similarly long time of evolution, one would expect a spread similar to the one of HBV/A1. As this is not the case, a long evolution of HBV genotype E in the general West African population is very unlikely. Thus historical migration patterns fully support both in our study and in the one by Kramvis and Paraskevis [36] the phylogenetic results obtained with BEAST.

Indeed, in the present analyses the 10 HBV/E S-gene strains from Haiti were interspersed within the African strains clustering with strains from Nigeria and Mali. They seemed to be the result of multiple introductions only after the HBV/E spread along the West African coast (Figure 1a), with an observed tMRCA of the monophyletic Haitian clades of 62.4 and 55.9 years based on the above substitution rate of 1.9×10^−4^ s/s/y. These recent introductions are supported by the low variability in the different HBV/E cluster from Haiti in the phylogenetic tree. In contrast to strains from Africa that form regional clusters, strains from Haiti do not cluster together, but seem to originate from different African cluster and even from different countries, supporting the conclusion that individual HBV/E strains would have been introduced to Haiti only recently. This, as well as the only rare strains of HBV/E detected in the Americas [11], indicates that HBV/E was introduced and also spread in the general West African population only recently and after the end of the transatlantic slave trade.

The only recent introduction of HBV/E into the general West African population is furthermore supported by Bayesian skyline analyses that indicate an extensive increase in effective numbers of HBV/E infections (Figure 3) and is indicative of a recent and rapid spread of the virus explaining today's hyperendemicity in this region.

Previous studies estimated substitution rates of 7.72×10^−4^ s/s/y for HBV in general [22] and 3.2×10^−4^ to 4.3×10^−4^ s/s/y when analyzing individual HBV genotypes [15], [22]. The latter substitution rates are comparable with those observed for HBV/E in our study. However, a recent study by Paraskevis et al. linked the evolution of HBV worldwide to human migratory patterns [23]. The authors suggest that HBV co-expanded and co-migrated with human populations within the last 34,000 years and estimated a long-term substitution rate of 2.2×10^−6^ s/s/y for HBV in general and a tMRCA for HBV/E in Africa to be 6000 years (95% HPD: 3200–9400 years).

When applying this substitution rate to our HBV/E S-gene dataset, we found a tMRCA of 3717 years. This is, however, nowhere close to the 130 years of evolution, or even the upper limit of the confidence interval of 441 years observed in our study and is also incompatible with the absence of HBV/E in other parts of the world.

While methodological differences, such as the use of the S-gene or the full-length genome or differences in datasets may explain some of this discrepancy, biological considerations are equally important. Paraskevis et al. base their analyses on a limited dataset of selected S-genes of all HBV sub-/genotypes in order to infer a long-term substitution rate. However, the S-gene region is highly constrained in terms of viral evolution, as it is fully overlapping with the viral polymerase gene. Such a conserved region may be appropriate for the study of long-term evolution, but not for HBV/E in Africa and for the estimation of (short-term) substitution rates. Because of the observed low genetic variability of HBV/E and because the S-gene does not fully represent the HBV variability (e.g. HBV genotypes and subgenotypes are defined on the basis of the full-length genome [10], [37]), our analyses on the full-length genome more closely reflect the time of evolution of HBV/E. Nevertheless, our results could at least be partially reconciled with those of Paraskevis et al. assuming that since its separation from the most closely related genotype D, HBV/E was confined for thousands of years to an isolated population. From there the virus would have spread to the general West African population most likely within the last 130 years, but certainly less than 400 years ago, as indicated by the upper limit of the confidence interval. However, where HBV/E was confined during these thousands of years before the spread is unclear.

Nevertheless, based on a tMRCA of 130 years, the most prominent increase of HBV/E infections would have occurred approximately until the 1930s (Figure 3). As HBV is transmitted not only sexually, but also through percutaneous or parenteral contact with infected blood and body fluids [1], an additional explanation for this phenomenon would be iatrogenic transmission in well intended mass-injection campaigns with unsafe injections by the colonial powers (as reviewed in [11]). These had previously been considered an important factor in the spread of hepatitis C virus (HCV) and human immunodeficiency virus (HIV) in this region [38], [39], [40]. As the more stable HBV is estimated to be 10 and 20 times more transmissible via unsafe injections than HCV and HIV, respectively [41], [42], such a transmission should be considered as an alternative explanation for such a rapid spread of HBV/E.

Efficient horizontal transmission would accelerate viral replication and contribute to the high substitution rate observed. While the rapid spread of HBV/E can be explained by efficient horizontal transmission whether sexually, by unsafe mass injection campaigns or other practices, the current high contemporary prevalence of chronic carriers would be indicative of a high infection rate during early childhood.

While our study cannot provide a clear origin of HBV/E in Western Africa, Bayesian MCMC analyses corroborate a massive transmission and rapid expansion of HBV/E in the last 130 years. This is supported by MJN and skyline analyses, which showed a rapid increase of HBV/E infections, approximately until the 1930s. These results are in line with the low genetic diversity, the high prevalence of HBV/E in Africa and its absence in other parts of the world, indicating that the hyperendemicity of HBV in Western Africa is a recent phenomenon and likely due to dramatic changes in the routes of transmission in a relatively recent past.
